# Seed Coating Increases Seed Moisture Uptake and Restricts Embryonic Oxygen Availability in Germinating Cereal Seeds

**DOI:** 10.3390/biology6020031

**Published:** 2017-05-24

**Authors:** Linda Gorim, Folkard Asch

**Affiliations:** 1 Department of Plant Sciences College of Agriculture and Bioresources, University of Saskatchewan, 51 Campus Drive, Saskatoon, SK S7N5A8, Canada; linda.gorim@usask.ca; 2 Institute for Agricultural Sciences in the Tropics (Hans-Ruthenberg-Institute), University of Hohenheim, Garbenstr. 13, 70599 Stuttgart, Germany

**Keywords:** barley, imbibition, invertase, oxygen profile, rye, sucrose metabolism, wheat

## Abstract

Seed coating is a technology to improve germination and homogenize stand establishment. Although coating often results in lower germination rates, seeds that do germinate grow more vigorously and show strongly reduced respiratory losses during reserve mobilization. We hypothesize that the higher mobilization efficiency is due to a shift in the enzymatic cleavage of sucrose from invertase to sucrose synthase in the embryonic tissue caused by a reduced oxygen availability induced by oversaturation with water caused by the coating during early germination. We investigated the effect of coating on barley, rye, and wheat seed imbibition during the first 30 h after seeds were placed in moisture. We profiled oxygen in the embryos and measured sucrose and acid invertase levels as imbibition progressed. We found that seeds within coatings absorbed significantly more moisture than uncoated seeds. Coating resulted in near anoxic oxygen concentrations in the developing embryonic tissues in all three species. In barley, sucrose was not cleaved via the invertase pathway, despite the fact that invertase activity in coated seeds was increased. In rye and wheat, invertase activities were significantly lower in embryos from coated seeds without significantly changing the sugar composition.

## 1. Introduction

Seed coating with hydro-absorbers as a technology to overcome water-related problems in drought-prone agricultural systems generally results in low or reduced germination rates [1,2,3,4]. Earlier studies attributed low germination rates to effects of the coating on water imbibition and available oxygen in the seed [5,6]. In contrast, in those seeds germinating, hydro-absorber coated barley, rye, and wheat seeds with coat shares greater than 75% of the average seed have been shown to promote better seedling growth compared to those seedlings growing from uncoated seeds [4] by increasing efficiency of grain reserve mobilization via a possible switch from invertase-based to sucrose synthase-based embryonic sucrose breakdown [7]. We have argued that this apparent switch in activity of sucrose metabolic enzymes could be due to a reduced oxygen supply to the embryo caused by a higher saturation of the seed with water supplied from the coating [7,8].

In general, germination progresses in three phases that are delineated by water uptake characteristics. Germination begins with rapid imbibition of water in phase I, followed by a plateau phase of seed moisture content in phase II, during which the enzymatic and energetic basis for reserve mobilization is laid [9,10,11]. Phase III begins with the radicle emergence and is characterized by a rapid increase in seedling water content and massive reserve mobilization from storage tissues. In an earlier study [7], we reported on early seedling growth and sucrose and glucose mobilization during phase III of germination. When growing from coated seeds, barley, rye, and wheat differed strongly in mobilization efficiency and thus, in the respiratory losses of reserves mobilized during germination as compared to seedlings growing from uncoated seed. This indicated a strong effect of the coating on the enzymatic breakdown of starch and sucrose during germination, suggesting a major influence on the seed metabolism during phase II of germination (the first 48 h of seed germination) when major enzymes involved in carbohydrate and protein metabolism are activated [11]. Carbohydrate metabolism has been shown to be active one hour after imbibition based on metabolite levels, suggesting an immediate increase in the activity of glycolysis and the Krebs cycle that facilitates early energy-demanding processes [8,12]. Transcripts and mRNAs encoding starch breakdown and several sucrose synthesizing and cleaving enzymes already present in dry seeds are activated during phase I and II of germination [12], with the level of activation (up- or downregulation) depending on environmental conditions and the physiological state of the seed [10]. The degradation of sucrose is a major pathway fueling glycolysis [13]. Sucrose can be cleaved by either invertase (into glucose and fructose) or by sucrose synthase into fructose and uridine diphosphoglucose, depending on tissue oxygen availability [13,14]. The recent development of oxygen-sensitive micro-sensors allows measuring oxygen profiles in living tissues. So far, research is focused mainly on developing and maturing seeds [15,16] and little is known about the oxygen distribution in tissues of germinating cereal seeds.

For the current study, we hypothesize that coating strongly affects water uptake to the seed, this in turn strongly influences the oxygen availability in tissues critical for successful mobilization of endosperm reserves which in turn has an effect on the activities of the sucrose cleaving enzymes, providing the developing embryo with the sugar required for growth. The focus of our study is, therefore, three pronged: (1) we investigated imbibition during the first 30 h in seeds of wheat, rye, and barley coated with the hydro-absorber, Stockosorb^®^ (Evonik Nutrition & Care GmbH, Krefeld, Germany) compared to that of the uncoated seed employing the widely and successfully used (e.g., [17,18]) imbibition model developed by Peleg [19]. (2) We determined the oxygen concentration in the various tissues of the germinating seed with and without coating. (3) We investigated sucrose and glucose abundance in early embryonic tissue with the aim to determine which enzymatic activity may govern sucrose metabolisms in either coated or uncoated seeds.

## 2. Materials and Methods

### 2.1. Plant Material and Treatments

Seeds of spring barley (*Hordeum vulgare* L., cv. Maltasia), winter rye (*Secale cereal* L., cv. Jobaro), and spring wheat (*Triticum aestivum* L., cv. Thasas) were obtained from Freudenberger Feldsaaten GmbH and used in all experiments. Seeds of all three cereal species were either uncoated or coated with a seed coating developed by Freudenberger Feldsaaten GmbH containing a specific amount of hydro-absorber (Stockosorb). The term “coated” refers to grains with a coat share greater than 75% of the total mass. The term “uncoated” refers to the original seed.

### 2.2. Determination of Grain Initial Moisture Content

The initial moisture content of the seed was determined following the standard procedure laid down by International Seed Testing Association (ISTA) [20]. For each cereal species, randomly selected 25 coated seeds, uncoated seeds, and kernels from within the coating (coated seeds of which the coating was removed) respectively were cut longitudinally using a sharp blade into halves and each half was then sliced four times. The sliced grains were immediately placed into 2 mL pre-weighed test tubes and weighed again. The samples were then transferred to an oven and dried at 103 °C for 17 h ± 15 min and later cooled in a desiccator for 1 h ± 15 min and weighed. The mass of the individual seeds was calculated by subtracting the mass of the empty test tubes from those of the test tube containing seeds. The amount of water lost was the difference between the weight in the pre-oven test tubes and post-oven test tubes. Therefore, the initial moisture content (MCi) was the moisture difference divided by the seed mass expressed in percentage.

### 2.3. Estimating Grain Moisture Content over Time

Twenty-five seeds were randomly selected for all cereals from all treatments and individually weighed on an electronic balance. Coated as well as uncoated seeds of each cereal were placed on moist filter paper (Ecolab-Bogen-Filterpapier, Neolab, Heidelberg, Germany) in labeled plates 19.5 cm by 19.5 cm and plates were immediately transferred to the growth chambers (Percival Intellus Ultra Controller) and maintained at 25 °C. Individual uncoated seeds and coated seeds with the coating attached and coated seeds with the coating removed were directly weighed every 3 h for the first 12 h and subsequently every 6 h until the 30th h. After each weighing time, the 25 coated seeds were quickly washed, blotted on filter paper to remove excess water, and weighed to get a weight of the seed within the coat. The difference between an individual grain weight at a given time *t* and its original weight at time *t*_0_ divided by its weight at time *t*_0_ expressed as a percentage plus MCi at time *t*_0_ gives the moisture content (MC) at that point in time. The MC for the seeds within the coatings was estimated based on the assumption that their initial weight was the same as that of an average seed (on the thousand grain weight bases).

The moisture content in the coating was calculated as:

1. Individual coated grain (coat share >75%) − average uncoated grain = weight of coating
2. Weight of imbibed grain − weight imbibed seed within = weight of imbibed coating
3. Weight of imbibed coating − weight of coating = amount of water
4. Moisture content of coating = (amount of water/Imbibed coating) × 100

### 2.4. Estimating Grain Imbibition Rate and Capacity

Knowing the initial MC and MC enabled the estimation of the imbibition rate and imbibition capacity from the model postulated by Peleg [19] based on a two-parameter sorption equation, thus:

(1) $$M_{t}\;=\;M_{0\;}\pm \;\frac{t}{K_{1}+\;K_{2}t}$$

where *M_t_* is the MC of grains at time t in percentage, *M*_0_ is the initial MC (%), *K*_1_ is the Peleg rate constant (min %^−1^), and *K*_2_ is the Peleg capacity constant (%^−1^). High values of *K*_1_ or *K*_2_ imply low imbibition rate (IR) and low capacity, respectively, and the inverse is true for low values of *K*_1_ and *K*_2_. Imbibition is an absorptive process and so ‘±’ in Equation (1) becomes ‘+’. The imbibition or absorption rate (R) was obtained from the first derivative of Equation (1):

(2) $$R=\;\frac{dM}{dt}\;=\;\pm \;\frac{K_{1}}{{\left(K_{1}+\;K_{2}\right)}^{2}}$$

The Peleg rate constant *K*_1_ is the inverse of the initial imbibition rate (IR) of grains (R_0_) at time *t*_0_. The Peleg imbibition capacity constant *K*_2_ relates to the maximum or minimum attainable grain MC. As *t* →∞, Equation (1) gives the relation between grain equilibrium moisture content (*M*_e_) and *K*_2_:

(3) $$M│t_{\infty }={\;M}_{e}=M_{0}\pm \;\frac{1}{K_{2}}$$

In order to obtain *K*_1_ and *K*_2_, Equation (1) was linearized resulting in the following Equation:

(4) $$\frac{t}{M_{t}-\;M_{0}}\;=\;K_{1}+\;K_{2}t$$

A plot of *t*/*M_t_* − *M*_0_ versus time, therefore, resulted in a straight line, with *K*_1_ being the y-intercept and *K*_2_ the slope, and from these constants, the equilibrium MC (*M*_e_) and initial IR (R_0_) were calculated. The water absorption capacity and IR between coated and uncoated seeds were estimated by comparing the slopes of the straight lines produced when Equation (4) was plotted. The imbibition rate was also expressed in terms of the amount of water imbibed by the grain per hour.

### 2.5. Measurement of Oxygen Concentrations in the Coated and Uncoated Seeds

**Figure 1:**
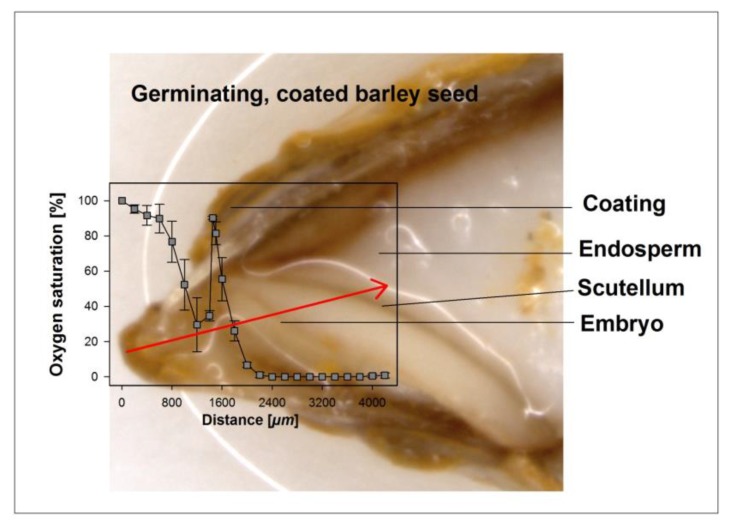
Exemplary illustration of an oxygen profile through the coating, the seed coat, and the embryo into the endosperm. The oxygen profile shown was taken along the path of the micro-probe, as indicated by the arrow on the image. Distances shown in the graph do not fully match dimensions in the image due to a slight skew in the longitudinal cut.

The oxygen concentrations across a section of uncoated seeds or seeds coated with hydro-absorber were determined for barley, rye, and wheat using a less than 50 µm tip glass fiber needle-type oxygen micro-sensor (micro-sensor, Presens, Regensburg, Germany) connected to a fiber optic oxygen meter (Microx TX3, Presens) with the sensor mounted on a micro-manipulator, as described by Rolletschek et al. [21]. Before measurements began, the oxygen sensor together with the temperature sensor was calibrated in oxygen free sodium sulfite (Na_2_SO_3_) dissolved in 100 mL distilled water and later in pure water, as recommended by the manufacturers. Coated and uncoated seeds were randomly selected and placed intermittently in 30 min intervals at marked positions on moist filter paper and immediately transferred to the growth chambers at 25 °C. After 18 h and then every 6 h, seeds were removed in the sequence in which they were placed in the plates, the coating was quickly washed off and excess water was blotted off. Oxygen profiles were measured by fixing the kernel on a micro-manipulator stage and driving the oxygen needle tip at 50, 100, and then 200 µm distances into the embryo from the top and through the scutellum with the aid of the micro-manipulator; the readings were allowed to stabilize and were recorded, after which the needle was driven to a new position and the process was repeated (Figure 1). The path of the needle was verified and confirmed by dissecting each seed at the end of each measurement and viewing under a microscope. In order to measure the amount of oxygen in the coating, the same process was repeated but the hydro-absorber coating was not washed off and the whole grain was mounted. To protect the needled tip, the whole capsule was moved into the coating and the needle tip extended only at the sites where measurements were required. The thickness of coating, testa, and endosperm were determined by observing at least five representative seeds at 10-fold magnification under a stereo microscope (LeitzBioMeD) calibrated with a stage micrometer scale. Data are presented as means of at least six grains and oxygen was recorded as percentage saturation.

### 2.6. Extraction and Quantification of Sugars

Barley, rye, and wheat seeds, both coated and uncoated, were place on moist filter paper in plates, as previously reported in [7]. Every 6 hours from the 12th h, the coating was washed off the coated seed and in both coated and uncoated seeds, embryonic material was quickly excised with a stainless steel razor blade on ice, and freeze dried in 2 mL Eppendorf vials.

Ten milligrams of freeze dried embryonic material per replicate were ground in ‘FastPrep Lysing matrix A’ tubes at 6.0 m per second for 40 s in the FastPrep^®^ (MP Biomedicals, Strasbourg, France)—24 homogenizer. Five hundred microliters (500 µL) 80% ethanol was added, the tubes vortex and heated at 60 °C for 30 min. Each tube was centrifuged for 10 min at 13,000× *g* and the supernatant transferred to another Eppendorf-vial. The process, from addition of ethanol to centrifugation, was repeated twice. The ethanol extract was evaporated to dryness in a vacuum centrifuge (Eppendorf Concentrator 5301, Eppendorf GmbH, Hamburg, Germany) at 45 °C for 2.5 h and re-dissolved in 900 µL water by shaking overnight. Sugars were assayed as described by Andersen et al. [22]. For glucose, 100 µL were assayed in a total volume of 200 µL water. The dye, glucose oxidase/peroxidase/ABTS-solution (400 µL), was added and samples were incubated in a water bath at 37 °C for 30 min.

For sucrose plus glucose, 50 µL were assayed in a total volume of 300 µL in NaAc-buffer containing 50 mM Na-acetate and 15 mM magnesium chloride (pH 4.6). Invertase solution, 2 µL, and the dye were added. Samples were read at 418 nm in a spectrophotometer (Beckmann, Type UV-Du640, Beckmann Coulter GmbH, Krefeld, Germany).

### 2.7. Calculation of the Amount of Sugars

From the linear standard curve, the amount of sugars in each sample measured per milligram dry matter or milligram reserves remobilized was calculated. For example, the amount of glucose per milligram sample (mg/mg) in an unknown sample:

(5) $$x=\frac{\left[0.3\;\mathrm{mL}\right]\left[Y_{1}\left(\frac{\mathrm{mg}}{\mathrm{mL}}\right)\right]\left[0.9\;\mathrm{mL}\right]}{\left[0.05\;\mathrm{mL}\right]\left[Z\;\mathrm{mg}\right]}$$

where: *x* is the amount of sugar in mg/mg dry weight plant material; *Y*_1_ (mg/mL) is the concentration of glucose obtained from the standard curve; 0.300 mL. [*Y*_1_ (mg/mL)] is the amount of glucose in 300 μL assayed (mg); 0.300 mL. [*Y*_1_ (mg/mL)]. [(0.900/0.05 mL)] is the amount of glucose in the total (900 μL) samples; *Z* mg can either be weight lost (∂) from grain or increase in dry matter (∂1).

### 2.8. Quantification of Enzymatic Activity

Crude enzyme extracts from approximately 10 mg of freeze dried and frozen material replicated three times were ground in ‘FastPrep^®^ Lysing matrix A’ tubes at speeds of 6.0 m·s^−1^ for 40 s in the FastPrep-24 machine. Five hundred microliters (µL) of extraction buffer consisting of 50 mM HEPES-NaOH, 1 mM EDTA, and 2.5 mM dithiothreitol, (pH 7.0) was added to the samples that were vortex. The samples were then centrifuged for 10 min at 20,000× *g* to pellet insoluble material. The soluble protein extract was removed and insoluble proteins extracted with buffer containing 1 M NaCl, 50 mM HEPES-NaOH, 1 mM EDTA, and 2.5 mM dithiothreitol, pH 7.0. Soluble protein extract (400 µL) was dialyzed against extraction buffer for 20 h at 0 °C on a 14,000 MWCO dialysis membrane (Visking, Karlsruhe, Germany) to remove endogenous soluble carbohydrates. The concentration of total protein was measured in the extract as described by Bradford [23] using a bovine serum albumin (BSA) standard.

Activities of soluble acid invertase and insoluble acid invertase were measured as described by Tsai et al. [24] with minor modifications. Invertase extracts (20 µL) were assayed in a total volume of 300 µL, with an assay buffer containing 50 mM sodium acetate, 15 mM magnesium chloride, and 100 mM sucrose (pH 5.0). Assays were incubated for 1 h at 30 °C, with blank terminated immediately after addition of protein extracts. All reactions were terminated by boiling and 400 µL glucose oxidase/peroxidase/ABTS-solution added to all tubes that were incubated at 37 °C in a water bath for 30 min. Samples were read in a spectrophotometer (Beckman, Type UV-Du640) at a wavelength of 418 nm. Three samples were assayed for each seed category with duplicate quantification of each.

### 2.9. Statistical Analysis

The difference in moisture content and total acid invertase activity between seeds from within the coating and uncoated seeds was calculated with the aid of the Proc GLM procedure of SAS. Least significant differences between means for moisture contents were calculated for time that measurements were carried out in all cereals, meanwhile, for differences in total acid invertase activity, letters were allocated with dissimilar letters on a pair of bars denoting significant difference at alpha equals 5%. The differences in the amount of water imbibed by both seeds from the coating and uncoated seeds for a given treatment and resultant parameter and between cereals was also carried out by Proc GLM procedure in SAS and letters were allocated to denote significance at alpha equals 5%.

## 3. Results

### 3.1. Effects of Coating on Imbibition

**Figure 2:**
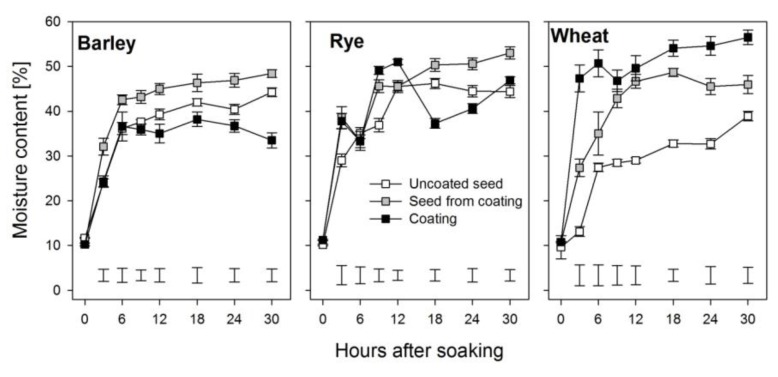
Moisture content of seeds within the coat, the uncoated seeds, and the coating during the first 30 h after soaking in barley, rye, and wheat. Error bars represent standard error of means and bars at the bottom of the graphs represent the least significant difference between treatments at each point in time.

In order to germinate, seeds imbibe water. The entry of water into seeds increases their moisture linearly which later plateaus at equilibrium moisture content [18,19]. This pattern was also observed in the current study for barley, rye, and wheat seeds. Barley and wheat seeds from within the coating had higher moisture contents compared to their uncoated counterparts (Figure 2). This was reflected by the high imbibition rate constant (*K*_1_) for seeds within the coating (SFC), while their imbibition capacity constant, *K*_2_, was consistently lower (Table 1). The initial absorption rate (R_0_) was higher in the coated seeds (entire grain) in all three cereal species as compared to the uncoated seeds, but the SFC had the lowest initial absorption rates (Table 1). The initial moisture content of the seeds within the coating was increased by coating in rye and wheat but not in barley. In comparison to the uncoated seeds, the equilibrium moisture content (*M*_e_) was increased in the seeds from within the coats in all three cereals, with the largest increase (15.5%) in wheat and the smallest (4.5%) in barley (Table 1).

### 3.2. Oxygen Profiles in Coated and Uncoated Seeds

**Figure 3:**
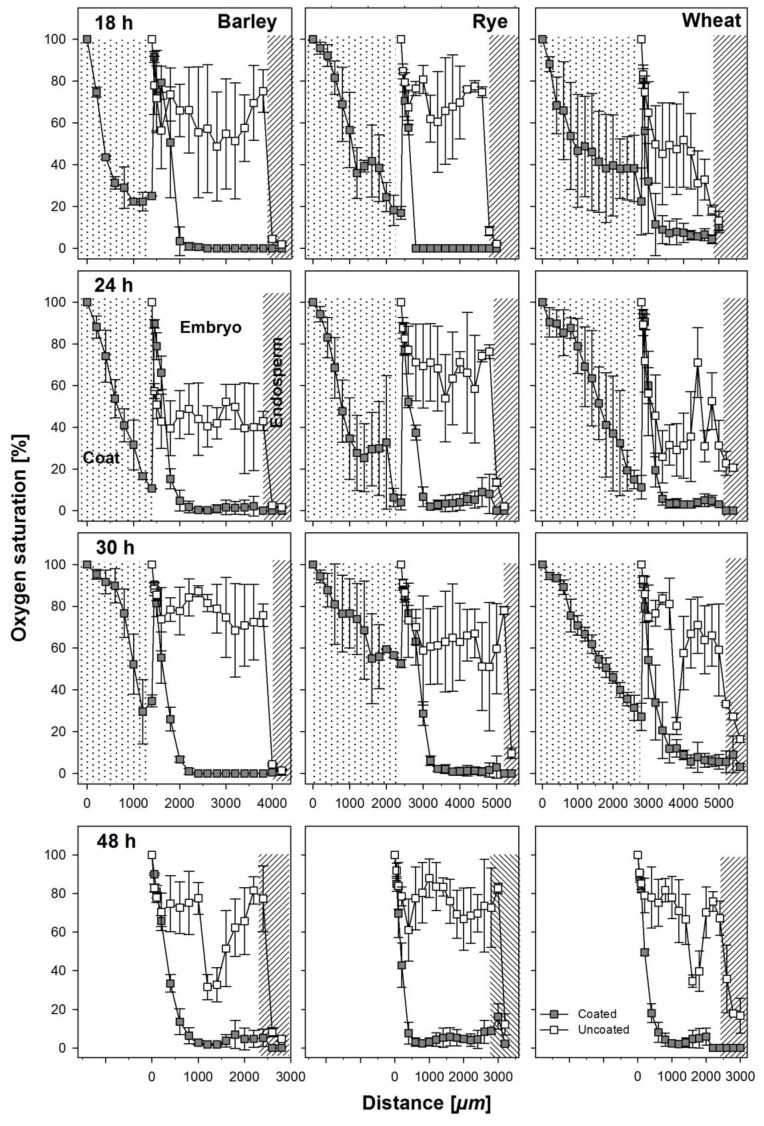
Oxygen profiles taken as indicated in Figure 1 at 18, 24, 30, and 48 h after soaking from coated and uncoated seeds of barley, rye, and wheat. The apparent increase of oxygen concentration after the coating material in the profiles from the coated seeds is an artifact due to the necessity to remove the coat before entering the micro-probe into the embryo. Coats where not present anymore at 48h after soaking and the radicula was visible. Error bars represent standard error of means.

Oxygen saturation levels were measured across coated and uncoated seeds of all three cereals through the embryonic tissue into the endosperm (Figure 1) at regular intervals during the first 48 h after soaking. The coating material was hard and could not easily be penetrated and because of this, we measured oxygen levels in two steps. The sensor was first inserted through the coating material until it reached the testa. Then, to avoid contaminations, the seed coating was washed off and the sensor was newly placed and inserted into the seed. Thus, the apparent increase of oxygen saturation between the coating and the seed in measurements from coated seeds is an artifact owed to the measuring technique (Figure 3). In general, oxygen saturation decreased rapidly in the coating of coated seeds by 80–90% of atmospheric oxygen levels and remained at levels of 1–5% in barley and rye embryos in seeds from within the coating and 8–10% in wheat embryos from seeds from within the coating (Figure 3). The three species differed substantially in the oxygen saturation levels in the embryo in the uncoated seeds during the first 30 h of germination. In barley, average oxygen saturation in the embryo varied between 50% and 80% of atmospheric levels; in rye, mean oxygen saturation was between 70% and 80%; and in wheat, oxygen saturation levels decreased with increasing distance into the embryo to levels of 20–30%, with mean values at about 40%. In barley and rye, endosperm oxygen saturation in coated and uncoated grains was between 1% and 10% whereas in wheat, endosperm oxygen saturation in uncoated seeds was at about 20% and in coated seeds between 1% and 5%. After 48 h, the radicle had already broken through and no more coating material was present at the site of sensor entry. Still, the embryonic oxygen saturation levels were strongly reduced to 1–5% in coated seeds as compared to uncoated seeds in all three species.

**Figure 4:**
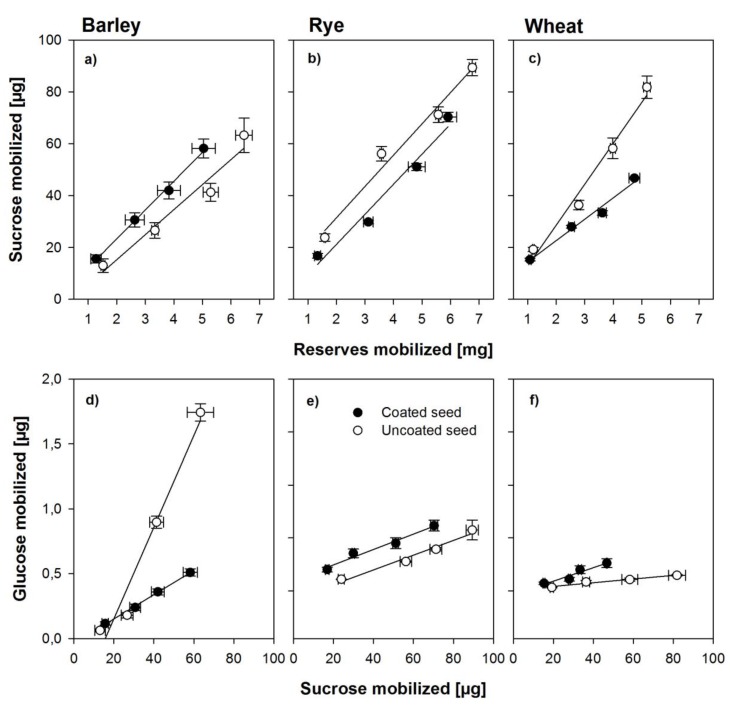
Relationships between the amount of reserves mobilized from the grain and the amount of sucrose found in the embryo (**a**–**c**) and the relationship between the amount of sucrose and glucose found in the embryonic tissues (**d**–**f**) in the seed from within the coat and uncoated seeds of barley, rye, and wheat. Error bars represent standard error of means.

Theoretically, sucrose in either endosperm or scutellum is synthesized from starch breakdown and should thus be linearly related with endosperm mobilization, whereas glucose found in the embryo should be a product of sucrose cleavage in the embryo. If sucrose is cleaved into anything else but glucose and fructose, for example, UDP glucose, the analyses used in this study would not detect it as glucose. Figure 4 shows the relationship between mobilization of endosperm reserves and sucrose found in the respective embryos and the relationship between this sucrose and glucose found in the same tissues during the first 30 h of germination. Sucrose in the embryo was linearly related to the amount of endosperm reserves mobilized in all three species and amounts were similar with minor differences between species (Figure 4a–c). For glucose as related to sucrose, the situation was different. The method of sugar analyses used here only detects glucose but not UDP-glucose. A strong linear relationship between glucose and sucrose mobilized was found in the embryos from uncoated seed, indicating an active invertase-based cleavage pathway for sucrose, whereas less than a third of glucose was found in the embryos from coated seeds, indicating an inhibition of the invertase-based pathway (Figure 4d). For the embryos from coated seeds in rye and wheat, a similar pattern was found (Figure 4e–f), although in rye and wheat embryos from coated seeds, smaller amounts of sucrose mobilized endosperm reserves were found when compared with the uncoated seed (Figure 4b–c). However, in these species, very little glucose was found in embryos from uncoated seeds, despite the fact that sucrose was amply available (Figure 4e–f).

### 3.3. Effects of Coating on Soluble and Insoluble Invertase Activity

**Figure 5:**
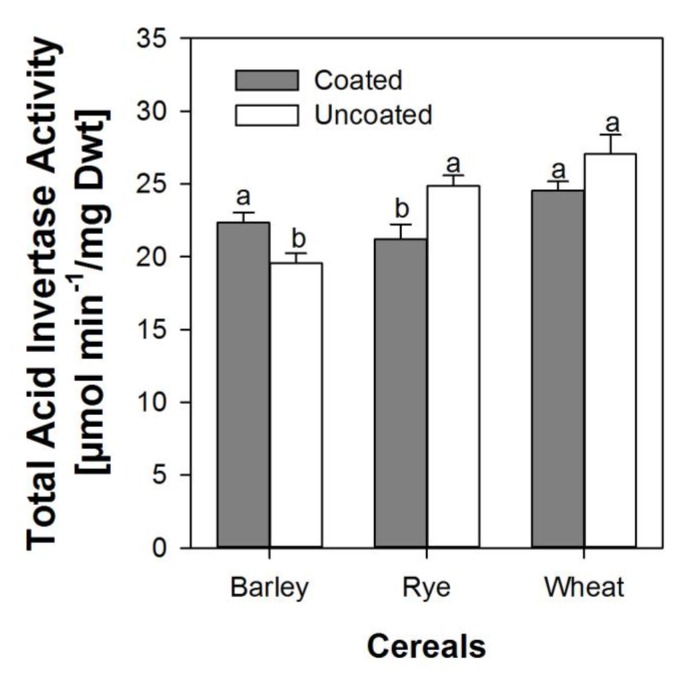
Total acid invertase activity averaged across the first 48 h after soaking in embryonic tissue from seeds from within the coat and uncoated seeds of barley, rye, and wheat. Error bars represent standard error of means. A bar pair with dissimilar letters is significantly different at alpha equals 5% and vice versa.

The activity of soluble and insoluble invertase in embryonic tissues of both coated and uncoated seeds did not show any specific significant trend at any specific point in time during the first 48 h. However, when combined and expressed as mean activity over 48 h, the total invertase activity was higher in barley embryos from coated seeds than in embryos from uncoated seeds, but significantly lower in embryos from coated seeds in rye and wheat (Figure 5).

## 4. Discussion

### 4.1. Effects of Coating on Water Imbibition

We studied water uptake of both coated and uncoated seeds for 30 h after imbibition. Imbibition begins during phase I of seed germination and continues through phase II [8]. Initially, there is a rapid uptake of water mainly through the micropyle of the dry seed (phase I) until all of the matrices and cell contents are fully hydrated [10,11,25]. This behavior was observed in both uncoated seeds and the seeds from within the coats of barley, rye, and wheat during imbibition; however, the seeds from within the coating showed a much steeper initial uptake, especially in wheat (Figure 2). This phase lasted for about 3 and 6 h in seeds from within the coating and uncoated barley and rye seeds, respectively, whereas duration of phase I was 6 and 12 h in wheat for uncoated seeds and seeds from within the coating, respectively. This delay in entering into phase II in wheat in the coated seeds may play a role in the delayed germination that was subsequently observed [4]. Although the moisture content of seeds from within the coating in these cereals was higher compared to that in the uncoated seeds, the imbibition rate was lower and the maximum imbibition capacity at any point was also lower in seeds from within the coating (Figure 2; Table 1). The reason for this could be that the water absorbed through the coating doted with hydro-absorber was channeled slowly to the kernel within, resulting in fewer membranes being damaged in the seeds enveloped by this coating compared to those in uncoated seeds, since it is known that rehydration imposes considerable stress upon cell components that may lead to leakage of solutes, which is indicative of temporal membrane damage [10,11].

Seed moisture content dynamics plotted to determine the imbibition rate and capacity showed similar patterns in both uncoated seeds and seeds from within coats with initial sharp increases in the amount of water absorbed which later level off at equilibrium with very high coefficients of determination (0.971–0.999), and this was in agreement with similar observations reported for other seeds [18,26]. However, the imbibition rate over time was increased in seeds for within the coat in barley and rye (Figure 2), resulting in more water absorbed into these seeds as imbibition progressed, which resulted in very high equilibrium moisture contents (Table 1). The difference in equilibrium moisture content between the seeds from within the coating and the uncoated seeds was 4.5%, 9.5%, and 15.5% for barley, rye, and wheat, respectively. The high equilibrium moisture content observed in rye and wheat could have led to oversaturation in the kernel within the hydro-absorber coating, resulting in low oxygen conditions, interruption of most metabolic processes—probably leading to tissue damage [10,25] and ultimately resulting in the poor germination rates reported in hydro-absorber coated wheat and, to a lesser extent, in coated rye [4].

### 4.2. Effect of Coating on Oxygen Availability

Oxygen concentrations in embryos have been investigated in maturing seeds with the aid of oxygen micro-sensors and, in some cases, reported in relation to the enzymes, invertase and sucrose synthase [15,16,21]. However, we investigated for the first time in situ the oxygen supply to the embryo in germinating seeds with the aid of oxygen micro-sensors. We measured oxygen concentrations through the center of the embryo passing through the scutellum in coated and uncoated seeds as well as in the hydro-absorber coating surrounding barley, rye, and wheat seeds at the site above the embryo. Measurements were done in the embryo because it is the site where products resulting from endosperm reserve mobilization are channeled to for seedling growth [27,28]. The presence of an imbibing hydro-absorber coating around the kernel resulted in strongly hypoxic conditions in the embryo. Relative to atmospheric conditions, oxygen concentration decreased in the hydro-absorber coating by 70% to 90%. Since the coating material was completely saturated with water, atmospheric oxygen was strongly inhibited to diffuse through this barrier to reach the seed within the coat. Thus, the coating and the water contained within effectively blocked the oxygen supply to the seed. Oxygen in the embryos of coated seeds was greatly reduced by 90–99% relative to atmospheric concentrations across species and time and about 45–75%, 60–70%, and 35–65% in barley, rye, and wheat, respectively, compared to their uncoated counterparts (Figure 3). However, oxygen measurements were carried out mostly in seeds, coated or uncoated, that would have germinated. Lower oxygen concentrations in the embryo do not imply that oxygen was completely lacking but that its amount was much reduced and with mobilization actively progressing, the amount available was quickly consumed. This was evident from oxygen profiles for tissues surrounding the embryo center which suggest that oxygen supply to and around the embryo vary depending on the position with respect to the embryo center and this also varied as imbibition progressed in both coated and uncoated seeds. The micropyle has been shown to be the main gateway through which water enters seeds [29] and may also serve as supplier of oxygen at the same time, since the endosperm of maturing cereal grains has been shown to be almost anoxic [15], as well as the endosperm of germinating grains, as shown here (Figure 3).

### 4.3. Effects of Coating on Sugar Metabolism and Enzyme Activity

Sucrose stored in the aleurone layer, and also produced from oil catabolism, is believed to be actively secreted into the endosperm and taken up by the embryo as an early energy source before starch breakdown becomes the dominant source of carbohydrate supply [30]. The oxygen concentration in the embryo affects the pathway of sucrose cleavage with hypoxic conditions resulting in inhibition of acid invertases and activation of sucrose synthase [13,14,31], whose level has been reported to be elevated in both barley and wheat embryos only but not at other sites [14]. This could easily show the importance of the pathway switch, since the reserves mobilized from the endosperm were proportional to the sucrose accumulation found in the embryo, thereby indicating no inhibition of the starch breakdown during germination resulting from low oxygen levels. However, the argument would hold only for both cereals (i.e., barley and wheat) if the glucose accumulated in the embryos of the uncoated seeds was proportional to the sucrose accumulated in the embryo, whereas no proportionality would be observed between glucose and sucrose accumulation in the embryonic tissues of seed from within the coat, which was the case for barley, but not for wheat (Figure 4). This may have resulted from different durations of the phasing during early germination, particularly imbibition and phase I [10,11], as described above, so the early germination phases may have not been fully aligned in relation to time, and consequently, sucrose transport or glycolysis in the embryonic tissue may have been delayed.

### 4.4. Effect of Hydro-Absorber Coating on Enzyme Activity

We determined the activities of soluble and insoluble invertase during the first 48 h after imbibition of all three cereal species for coated and uncoated seeds. Both activity as well as amounts present in the embryonic tissue did not vary systematically between species or coatings. Mean total invertase activity did show significant differences between species and coatings (Figure 5), however, it was not conclusive with regard to glucose and sucrose availability in the embryo. It is possible that we were not able to capture the depression of invertase activity that may have happened in vivo under strongly hypoxic conditions in the embryo, as the activity measurements were performed in vitro under laboratory conditions with oxygen present during the analyses. The mean values we found are in contrast to the report by Guglielminetti et al. [32], who reported very low invertase activity in barley and rye under anoxic conditions. We suggest that based on our knowledge of oxygen supply in the embryo, invertase was present but its activity was depressed, or, alternatively, both invertase and sucrose synthase might have been active at different times during imbibition.

## 5. Conclusions

As postulated earlier [7] we investigated moisture uptake to the uncoated seed and the seed within the coating as well as the oxygen profiles in the embryos of the respective seeds. We found that seeds within coatings absorbed significantly more moisture than uncoated seeds. Using a micro probe technology, we provided evidence that coating results in near anoxic oxygen concentrations in the developing embryonic tissues in all three species. In barley, sucrose was not cleaved via the invertase pathway anymore, despite the fact that invertase activity levels in coated seeds were increased. In rye and wheat, clear evidence from the sugar availability in the embryo could not be found, but invertase activities were significantly lower in embryos from coated seeds in these two species. In addition to genetic differences between the species, differences in the timing of imbibition and progressing germination may also have interfered with the measurements during the first 30 h of germination. The data on the enzymatic activities remained elusive, since within the experimental set-up it was not possible to measure enzymatic activities or abundance in situ. We could show that the functionality of the invertases was not compromised by the low oxygen environment. However, whether or not the activity is actually depressed within the living tissue remains the objective of further studies.

## Figures and Tables

**Table 1:** Imbibition characteristics during the first 30 h after soaking of coated and uncoated barley, rye, and wheat seeds. SFC refers to the seed within the coating; Uncoated refers to the original seed without coating. *K*_1_ is the Peleg rate, *K*_2_ the capacity constant, IC is the Imbibition capacity, and *M*_e_ is the equilibrium or saturation moisture content. Small letters refer to mean comparisons between SFC and the uncoated seed for each parameter analyzed at alpha equals 5%, meanwhile capital letters denote differences between cereals for the same treatment for any given parameter at alpha equals 5%.

Cereals	Treatments	*K*_1_ × 10^−2^ (h %^−1^)	*K*_2_ × 10^−2^ (%^−1^)	IC (%)	*M*_e_ (%)
**Barley**	SFC	5.0 (±0.8)^aB^	2.5 (±0.1)^aB^	40.4 (±0.8)^aB^	51.1 (±0.8)^aB^
	Uncoated	5.9 (±1.2)^aB^	2.8 (±0.1)^aB^	35.8 (±1.7)^aA^	47.5 (±1.7)^aA^
**Rye**	SFC	8.9 (±0.9)^aA^	2.1 (±0.1)^bC^	47.1 (±2.1)^aA^	58.3 (±2.1)^aA^
	Uncoated	5.4 (±0.3)^bB^	2.6 (±0.2)^aB^	37.9 (±0.3)^bA^	48.1 (±0.3)^bA^
**Wheat**	SFC	2.5 (±0.6)^bB^	2.8 (±0.1)^bA^	36.2 (±1.0)^aB^	47.0 (±1.0)^aB^
	Uncoated	11.8 (±2.1)^aA^	4.1 (±0.2)^aA^	24.3 (±0.9)^bB^	33.9 (±0.9)^bB^
